# Trunk-to-peripheral fat ratio predicts a subsequent blood pressure in normal-weight pubertal boys: a 3-year follow-up of the Kitakata Kids Health Study

**DOI:** 10.1186/s12199-020-00878-1

**Published:** 2020-08-20

**Authors:** Katsuyasu Kouda, Masayuki Iki, Yuki Fujita, Harunobu Nakamura, Masami Hamada, Kazuhiro Uenishi, Mari Miyake, Toshimasa Nishiyama

**Affiliations:** 1 Department of Hygiene and Public Health, Kansai Medical University, 2-5-1 Shin-machi, Hirakata, Osaka, 573-1010 Japan; 2 Department of Public Health, Kindai University Faculty of Medicine, 377-2 Oono-Higashi, Osaka-Sayama, Osaka, 589-8511 Japan; 3 Department of Health Promotion and Education, Graduate School of Human Development and Environment, Kobe University, 3-11 Tsurukabuto, Nada, Kobe, Hyogo 657-8501 Japan; 4 Department of Nursing, Chukyo Gakuin University, 2216 Toki-cho, Mizunami, Gifu, 509-6192 Japan; 5 Laboratory of Physiological Nutrition, Kagawa Nutrition University, 3-9-21 Chiyoda, Sakado, Saitama, 350-0288 Japan

**Keywords:** Body fat distribution, Blood pressure, Children, Densitometry, Epidemiology

## Abstract

**Background:**

Limited evidence exists regarding the relationship between central-to-peripheral fat ratio measured by dual-energy X-ray absorptiometry (DXA) and subsequent cardiometabolic risk in both pediatric and adult populations.

**Methods:**

The present cohort study investigated the relationship between DXA-measured body fat distribution and cardiometabolic parameters. The source population was 275 4th–6th graders (aged 9.6–12.6 years) in the northeast region of Japan (Shiokawa area in Kitakata). A 3-year follow-up was conducted to obtain complete information from 155 normal-weight children (87 boys and 68 girls). Normal-weight children were identified using sex- and age-specific international cut-offs for body mass index (BMI) based on adult BMI values of 25 kg/m^2^ and 18.5 kg/m^2^, respectively. Body fat distribution was assessed using the trunk-to-appendicular fat ratio (TAR) and trunk-to-leg fat ratio (TLR) measured by DXA.

**Results:**

In boys, systolic blood pressure (SBP) at follow-up showed a significant relationship with TAR at baseline after adjusting for age, height, pubic hair appearance, SBP, and whole body fat at baseline (*β* = 0.24, *P* < 0.05), and SBP also showed a significant relationship with TLR after adjusting for confounding factors including whole body fat (*β* = 0.25, *P* < 0.05). In girls, there were no significant relationships between blood pressure and TAR/TLR.

**Conclusion:**

Body fat distribution in normal-weight boys predicted subsequent blood pressure levels in adolescence. The relationship between fat distribution and blood pressure was independent of fat volume.

## Background

Excessive body fat accumulation is typically referred to as obesity and can impair health. Weight-based indices, such as body weight and body fat mass, are useful tools for assessing cardiometabolic risks in both children and adults [1]. On the other hand, some individuals are not obese by body weight, but are predisposed to type 2 diabetes, hypertension, and premature coronary heart disease [2]. Such normal-weight individuals with cardiometabolic risks are very common in the general population [2]. These individuals likely have significant excess visceral adipose tissue and are often referred to as metabolically obese, normal-weight (MONW) individuals [3, 4]. Visceral adiposity, rather than subcutaneous fat, has been considered to play a key role in cardiometabolic disease [5, 6]. However, individuals with high visceral fat tend to also have high subcutaneous fat, and this strong correlation between visceral fat and subcutaneous fat [7] interferes with the assessment of the independent effect of each regional fat mass on cardiometabolic disease in epidemiological studies.

Weight-independent indices such as waist-to-hip circumference ratio have been found to predict cardiometabolic risk factors [8–11]. Since waist circumference includes both the visceral and subcutaneous fats and hip circumference mainly includes subcutaneous fat, the waist-to-hip circumference ratio reflects visceral fat mass [8, 10]. The waist-to-hip circumference ratio may account for the characteristics of MONW individuals who suffer from metabolic complications [4]. However, as the waist-to-hip circumference ratio is an indirect index of adiposity, it cannot distinguish fat mass from fat-free mass and bone mass.

Dual-energy X-ray absorptiometry (DXA) allows for precise and accurate measurement of fat mass in the whole body and each body region (arm, leg, and trunk) [12]. DXA scanning was primarily developed for the diagnosis of osteoporosis, and its use has been extended to soft tissue composition measurement with low radiation exposure [12]. Trunk-to-peripheral fat ratio measured by DXA is a weight-independent parameter that can serve as an index of body fat distribution [13–21]. Trunk fat includes both visceral and subcutaneous fat, whereas peripheral (i.e., arm or leg) fat does not include visceral fat. Thus, the ratio of trunk-to-extremity (peripheral) fat has been reported to be a good indicator of visceral fat [15] and may reveal the relative impact of visceral to subcutaneous fat with regard to cardiometabolic risks [21]. Trunk-to-peripheral fat ratio measured by DXA may be able to characterize the profiles of MONW individuals.

Trunk-to-peripheral fat ratio (i.e., the trunk-to-leg fat ratio [TLR] and trunk-to-appendicular fat ratio [TAR]) measured by DXA at age 11 has been reported to predict blood pressure (BP) at age 14 in community-dwelling children in the central region of Japan (Hamamatsu City, Shizuoka), and that it is also meaningful for assessing body fat, particularly in underweight children [22]. Even among those who are not overweight or obese, fat distribution measured by DXA seems to be associated with cardiometabolic risk factors in childhood [23]. It may be important to consider body fat distribution in addition to body fat mass to assess cardiometabolic risks [23]. However, subjects in the previous study conducted in Hamamatsu had a narrower age range (11 years old at baseline) [22]. In addition, this study was unable to obtain data on potential confounders such as dietary intake and exercise [22]. Currently, no other cohort studies have examined DXA-measured trunk-to-peripheral fat ratio in relation to subsequent cardiometabolic risk in pediatric and adult populations, or whether it can serve as a valuable early indicator of cardiometabolic risk in childhood [23]. The present cohort study investigated the relationship between DXA-measured trunk-to-peripheral fat ratio at age 9.6–12.6 and cardiometabolic parameters at age 12.3–15.3 in normal-weight children in the northeast region of Japan (Kitakata City, Fukushima), adjusting for confounding factors including whole body fat.

## Methods

### Study population

The present study is part of the Kitakata Kids Health Study, which has been conducted every 3 years since 2001 in the Shiokawa area of Kitakata City, Fukushima, Japan [24, 25]. The city is located in the northeast region of Japan (139° east longitude and 37° north latitude). The source population was all 4th–6th graders (275 students: 148 boys and 127 girls; age range, 9.6–12.6 years) who were enrolled in four public elementary schools in Shiokawa in November 2013. As there are no other schools in this area, almost all children living in the area are enrolled in these schools. Among the 275 students, 255 (136 boys and 119 girls) participated in the baseline survey in November 2013 and provided information for DXA-measured body fat parameters and confounding factors. A follow-up survey was conducted in July 2016 to obtain information on BP and blood samples. We obtained complete information at both baseline and follow-up surveys from 218 students (followed-up population, 99 girls and 119 boys). Normal-weight children (study population, 68 girls and 87 boys) were identified from the followed-up population using body mass index (BMI) cut-offs at the baseline survey. Body weight, height, and waist circumference were measured in light clothing with no shoes. BMI (kg/m^2^) was calculated as weight (kg) divided by height squared (m^2^). Overweight and underweight children were identified using sex- and age-specific international cut-offs of BMI based on adult BMI values of 25 kg/m^2^ and 18.5 kg/m^2^, respectively. This determination was made using the standardized centile curves of BMI [26, 27].

### Measurement of whole body and regional fat

Whole body and regional fat parameters were determined with a single DXA scanner (QDR-4500A; Hologic Inc., Bedford, MA, USA) mounted on a mobile examination car at each elementary school. A single experienced medical radiology technician performed and analyzed scans for all participants. Quality control checks of the DXA scanner were completed on a daily basis using the step phantom. Participants were asked to wear light clothing, remove all metal objects from their body (jewelry, belts, snaps, underwire bras), and lay down on their back on the scanner table. After scanning was completed, a total-body posterior-anterior scan image was obtained. Using the total-body image, scan analysis for each regional body fat was performed. Arm, leg, and head regions were isolated from the trunk region using the following standard manufacturer-recommended methods [22, 28]: (a) the head region was distinguished from the trunk region with the horizontal shoulder line just below the chin, (b) arm regions were detected by the vertical shoulder line bisecting the shoulder joints, and (c) leg regions were separated from the trunk region including the pelvic region using angled cut lines bisecting both femoral necks. Two angled lines and a horizontal line just above the iliac crest defined the pelvic-triangle area in a posterior-anterior body image. TAR and TLR were calculated as ratios of trunk-to-peripheral fat [20]. Appendicular fat was calculated as the sum of arm fat and leg fat. TAR was calculated as trunk fat divided by appendicular fat, and TLR as trunk fat divided by leg fat.

### Measurement of BP and blood samples

Before blood sampling, a single experienced physician measured systolic blood pressure (SBP) and diastolic blood pressure (DBP) using an automated device (BP-103i2; OMRON COLIN, Tokyo, Japan) with an appropriate cuff size based on the upper arm circumference of each participant [29]. BP measurement was performed with participants in a quiet, sitting position for 5 min, with their back and left arm supported and both feet on the floor, at an appropriate ambient temperature as previously described [22]. The mean value of the two measurements was used for analysis. Non-fasting blood samples were collected by nurses. Total cholesterol (T-C) in serum was measured enzymatically using a commercially available kit (T-CHO-P·KL; Sysmex Co. Ltd., Kobe, Japan). Low-density lipoprotein cholesterol (LDL-C) and high-density lipoprotein cholesterol (HDL-C) in serum were measured by the direct method using commercial kits (Cholestest LDL and Cholestest N HDL; Sekisui Medical Co. Ltd., Tokyo, Japan). Hemoglobin A1c (HbA1c: National Glycohemoglobin Standardization Program) in the whole blood was measured by latex-agglutination test using a commercial kit (RAPIDIA® Auto HbA1c-L; Fujirebio Inc., Tokyo, Japan).

### Food frequency questionnaire (FFQ) and other questionnaire surveys

Dietary nutrient intake was estimated using a previously validated FFQ [30]. The questionnaire includes 3 to 5 grades of the frequency of food intake for each food item. Participants with parents/guardians chose one of the grades in the questionnaire beforehand. Responses to the questionnaire were verified by trained dieticians during interviews with participants. Intakes of nutrients and energy were estimated by the grade of frequency multiplied by relevant coefficients based on Japanese food composition.

Information regarding pubic hair appearance, time spent doing exercise, and time spent doing sedentary activities (e.g., watching television, playing video games, using a mobile phone, and using a computer) was also obtained using a self-reported questionnaire comprising multiple-choice single-answer questions. Participants with parents/guardians were allowed to choose only one of the predefined options (pubic hair appearance: 3rd grade, 4th grade, 5th grade, 6th grade, or no appearance; exercise: < 1 h/day, ≥ 1 and < 1.5 h/day, ≥ 1.5 and < 2 h/day, or ≥ 2 h/day; sedentary behavior: < 1 h/day, ≥ 1 and < 2 h/day, ≥ 2 and < 3 h/day, ≥ 3 and < 4 h/day, ≥ 4 and < 5 h/day, or ≥ 5 h/day). Their responses were verified by trained health care nurses through interviews.

### Statistical analysis

To assess sample bias created by drop-out cases during the 3-year follow-up period, comparisons between baseline characteristics of followed-up and dropped-out populations at the baseline survey were performed using unpaired *t* test or Mann-Whitney *U* test. Analysis of variance was used to evaluate differences in TAR/TLR and blood pressure among groups stratified by age. Simple linear regression analysis was performed to assess trends in TAR/TLR and blood pressure from the lowest to highest age group. For normal-weight children, simple regression analysis was used to examine the relationship between body fat parameters at baseline (independent variables) and cardiometabolic parameters at follow-up (dependent variables). To identify confounding variables which may affect relationships between TAR/TLR (predictors) at baseline and SBP at follow-up (outcome), Pearson’s correlation coefficients of potential confounding factors with main predictors and of potential confounding factors with outcome were calculated. Redundant variables unrelated to the main predictors and outcome in the bivariate analysis were excluded from the multivariate analysis. Relationships between parameters of fat distribution at baseline and BP at follow-up were evaluated using multiple regression analysis after adjusting for age, height, pubic hair appearance, whole body fat, and SBP/DBP at baseline. Mean values in each tertile of TLR were calculated from the general linear model after adjusting for confounding factors. For trend tests of the adjusted mean of BP at follow-up, multiple linear regression analysis was performed. The dependent variable was SBP at follow-up, and independent variables were tertiles of TLR, age, height, pubic hair appearance, whole body fat, and SBP at baseline. *P* < 0.05 was considered statistically significant. SPSS Statistics Desktop for Japan, Version 22 (IBM Japan, Ltd., Tokyo, Japan) was used for all analyses.

## Results

Table 1 shows baseline characteristics of the normal-weight population forming part of the followed-up population, as well as differences in the characteristics between the followed-up and dropped-out populations. No significant differences were observed in anthropometric parameters, pubic hair appearance, dietary intake, exercise, sedentary behavior, and body fat parameters between the followed-up and the dropped-out populations, except for SBP and the underweight proportion of boys. Table 2 shows baseline values of fat distribution parameters and blood pressure stratified by age. TAR and TLR in boys showed significant increases from the lowest to highest age group. SBP in girls also showed significant increases from the lowest to highest age group.

**Table 1:** Baseline characteristics of subjects

	Normal-weight population (study population)		Followed-up population		Dropped-out population		*P* values for difference^a^	
	Girls	Boys	Girls	Boys	Girls	Boys	Girls	Boys
	(*N* = 68)	(*N* = 87)	(*N* = 99)	(*N* = 119)	(*N* = 20)	(*N* = 17)		
Age (years)	11.1 ± 0.9	11.2 ± 0.9	11.1 ± 0.9	11.2 ± 0.9	11.3 ± 0.8	11.3 ± 0.7	ns	ns
Height (cm)	145 ± 8	144 ± 10	145 ± 9	143 ± 10	146 ± 9	140 ± 9	ns	ns
Weight (kg)	37 ± 7	36 ± 7	39 ± 9	39 ± 10	41 ± 13	34 ± 10	ns	ns
Body mass index (kg/m^2^)	18 ± 2	17 ± 1	18 ± 3	19 ± 4	19 ± 4	17 ± 4	ns	ns
Waist circumference (cm)	64 ± 5	63 ± 5	66 ± 8	66 ± 10	66 ± 12	62 ± 10	ns	ns
Overweight^b^, *N* (%)	N/A	N/A	19 (19)	25 (21)	3 (15)	3 (18)	ns	ns
Underweight^b^, *N* (%)	N/A	N/A	12 (12)	7 (6)	4 (20)	4 (24)	ns	0.01
SBP (mmHg)	98 ± 10	97 ± 9	100 ± 11	99 ± 10	101 ± 12	93 ± 10	ns	0.04
DBP (mmHg)	53 ± 7	50 ± 6	54 ± 7	51 ± 7	53 ± 9	50 ± 6	ns	ns
Pubic hair appearance, *N* (%)	28 (41)	16 (18)	43 (43)	22 (18)	10 (50)	1 (6)	ns	ns
Total energy intake (kcal/day)	2056 ± 323	2179 ± 498	2058 ± 363	2208 ± 465	1978 ± 319	2133 ± 384	ns	ns
Carbohydrate intake (g/day)	249 ± 49	268 ± 90	249 ± 54	271 ± 84	241 ± 64	260 ± 56	ns	ns
Protein intake (g/day)	92 ± 15	97 ± 21	91 ± 17	99 ± 20	92 ± 12	97 ± 19	ns	ns
Lipid intake (g/day)	80 ± 15	83 ± 14	80 ± 16	84 ± 14	74 ± 12	81 ± 17	ns	ns
Salt intake (g/day)	11 ± 2	11 ± 2	11 ± 2	11 ± 2	12 ± 2	12 ± 2	ns	ns
Exercise (e.g., jogging or sports), *N* (%)							ns	ns
< 1 h/day	40 (59)	35 (40)	59 (60)	52 (44)	13 (65)	6 (35)		
≥ 1 and < 1.5 h/day	24 (35)	41 (47)	32 (32)	54 (45)	7 (35)	8 (47)		
≥ 1.5 h/day	4 (6)	11 (13)	8 (8)	13 (11)	0 (0)	3 (18)		
Sedentary behavior (e.g., media use), *N* (%)							ns	ns
< 1 h/day	12 (18)	12 (14)	16 (16)	15 (13)	4 (20)	3 (18)		
≥ 1 and < 2 h/day	27 (40)	35 (40)	39 (39)	46 (39)	11 (55)	6 (35)		
≥ 2 h/day	29 (43)	40 (46)	44 (44)	58 (49)	5 (25)	8 (47)		
Trunk fat (kg)	2.7 ± 1.0	2.0 ± 0.7	3.0 ± 1.6	2.7 ± 1.9	3.5 ± 2.8	2.2 ± 1.9	ns	ns
Arm fat (kg)	1.2 ± 0.4	0.9 ± 0.3	1.3 ± 0.6	1.2 ± 0.8	1.5 ± 1.0	1.0 ± 0.7	ns	ns
Leg fat (kg)	3.8 ± 1.1	2.9 ± 1.0	4.0 ± 1.6	3.5 ± 2.0	4.4 ± 2.2	2.8 ± 1.9	ns	ns
Whole body fat (kg)	8.4 ± 2.6	6.6 ± 2.0	9.1 ± 3.8	8.3 ± 4.6	10.1 ± 6.0	6.8 ± 4.6	ns	ns
Whole body fat percentage (%)	22.0 ± 4.1	18.1 ± 4.8	22.7 ± 5.5	20.4 ± 7.1	23.6 ± 5.7	18.8 ± 7.4	ns	ns
TAR	0.53 ± 0.07	0.53 ± 0.08	0.56 ± 0.08	0.55 ± 0.09	0.56 ± 0.11	0.56 ± 0.10	ns	ns
TLR	0.70 ± 0.10	0.70 ± 0.11	0.74 ± 0.12	0.74 ± 0.15	0.74 ± 0.17	0.76 ± 0.15	ns	ns

*N* number, *ns* not significant, *SBP* systolic blood pressure, *DBP* diastolic blood pressure, *TAR* trunk-to-appendicular fat ratio *TLR* trunk-to-leg fat ratio

TAR was calculated as trunk fat divided by appendicular fat. TLR was calculated as trunk fat divided by leg fat. Values represent mean ± standard deviation, or N (percentage)

^a^*P* value calculated from the unpaired t-test or Mann-Whitney U test to compare followed-up and dropped-out populations. *P* < 0.05 was considered statistically significant

^b^Determined using age- and sex-specific body mass index cut-off points

**Table 2:** Baseline values of fat distribution parameters and blood pressure stratified by age

	TAR		TLR		SBP		DBP	
	Girls, *N* = 68	Boys, *N* = 87	Girls, *N* = 68	Boys, *N* = 87	Girls, *N* = 68	Boys, *N* = 87	Girls, *N* = 68	Boys, *N* = 87
Age groups								
9.65–10.62 years	0.53 ± 0.06	0.50 ± 0.07	0.69 ± 0.08	0.66 ± 0.10	92.8 ± 8.3	93.1 ± 8.7	51.9 ± 5.7	49.5 ± 7.1
10.64–11.63 years	0.52 ± 0.07	0.50 ± 0.05	0.67 ± 0.10	0.66 ± 0.08	102.1 ± 9.4	98.3 ± 8.7	55.8 ± 8.3	51.4 ± 6.1
11.64–12.64 years	0.56 ± 0.08	0.57 ± 0.08	0.74 ± 0.11	0.75 ± 0.11	101.0 ± 9.8	98.2 ± 10.2	52.8 ± 6.4	50.3 ± 5.9
*P* values for difference^a^	ns	<0.001	ns	<0.001	0.001	ns	ns	ns
*P* values for trend^b^	ns	<0.001	ns	<0.001	0.004	0.043	ns	ns

*TAR* trunk-to-appendicular fat ratio, *TLR* trunk-to-leg fat ratio, *SBP* systolic blood pressure, *DBP* diastolic blood pressure, *N* number, *ns* not significant

TAR was calculated as trunk fat divided by appendicular fat. TLR was calculated as trunk fat divided by leg fat. Values represent mean ± standard deviation

^a^Analysis of variance was used to evaluate differences among age groups

^b^Simple linear regression analysis was performed for trend tests from the lowest to highest age group. *P* < 0.05 was considered statistically significant

Table 3 shows the relationships between body fat parameters at baseline and cardiometabolic parameters at follow-up using simple regression analysis. In normal-weight boys, both TAR and whole body fat were significantly and positively related to SBP. Moreover, TLR in normal-weight boys was significantly and positively related to SBP.

**Table 3:** Relationships between body fat parameters at baseline and cardiometabolic parameters at follow-up

	SBP		DBP		T-C		LDL-C		HDL-C		HbA1c	
	*β*	*P*	*β*	*P*	*β*	*P*	*β*	*P*	*β*	*P*	*β*	*P*
Girls, N = 68												
Body mass index	0.11	ns	0.06	ns	− 0.01	ns	0.03	ns	− 0.10	ns	0.08	ns
Waist circumference	0.06	ns	0.06	ns	− 0.03	ns	0.02	ns	− 0.18	ns	0.04	ns
Whole body fat	0.10	ns	0.05	ns	0.10	ns	0.13	ns	− 0.05	ns	0.07	ns
Whole body fat percentage	− 0.02	ns	− 0.04	ns	0.24	0.05	0.28	0.02	− 0.01	ns	− 0.02	ns
TAR	0.02	ns	0.02	ns	0.00	ns	0.00	ns	− 0.14	ns	0.04	ns
TLR	0.00	ns	− 0.01	ns	0.04	ns	0.04	ns	− 0.14	ns	0.03	ns
Boys, N = 87												
Body mass index	0.29	< 0.01	0.11	ns	0.18	ns	0.14	ns	0.13	ns	− 0.36	< 0.01
Waist circumference	0.41	< 0.01	0.13	ns	0.24	0.03	0.22	0.04	0.09	ns	− 0.35	< 0.01
Whole body fat	0.25	0.02	0.01	ns	0.25	0.02	0.22	0.04	0.12	ns	− 0.31	< 0.01
Whole body fat percentage	0.00	ns	− 0.17	ns	0.17	ns	0.12	ns	0.11	ns	− 0.19	ns
TAR	0.27	0.01	0.20	ns	− 0.16	ns	− 0.09	ns	− 0.11	ns	0.16	ns
TLR	0.27	0.01	0.18	ns	− 0.18	ns	− 0.11	ns	− 0.11	ns	0.17	ns

*SBP* systolic blood pressure, *DBP* diastolic blood pressure, *T-C* total cholesterol, *LDL-C*, low-density lipoprotein cholesterol, *HDL-C* high-density lipoprotein cholesterol, *HbA1c*, hemoglobin A1c, *ß* standardized regression coefficient, *N* number, *ns* not significant, *TAR* trunk-to-appendicular fat ratio, *TLR* trunk-to-leg fat ratio

TAR was calculated as trunk fat divided by appendicular fat. TLR was calculated as trunk fat divided by leg fat. Simple regression analysis was used to examine relationships between cardiometabolic parameters (dependent variables) and body fat parameters at baseline (independent variables). *P* < 0.05 was considered statistically significant

Table 4 shows potential confounding variables which may affect relationships between TAR/TLR (predictors) at baseline and SBP at follow-up (outcome). Age, height, and public hair appearance were significantly related to TAR/TLR in boys. In addition, age, height, whole body fat, and SBP at baseline were significantly related to SBP at follow-up in boys.

**Table 4:** Potential confounding variables which may affect relationships between predictors and outcomes

	Predictors at baseline								Outcomes at follow-up							
	Girls, *N* = 68				Boys, *N* = 87				Girls, *N* = 68				Boys, *N* = 87			
	TAR		TLR		TAR		TLR		SBP		DBP		SBP		DBP	
	*r*	*p*	*r*	*p*	*r*	*p*	*r*	*p*	*r*	*p*	*r*	*p*	*r*	*p*	*r*	*p*
Potential confounding factors at baseline																
Age	0.17	ns	0.15	ns	0.34	0.01	0.30	< 0.01	0.39	< 0.01	0.23	ns	0.29	0.01	0.24	0.02
Height	0.22	ns	0.19	ns	0.35	< 0.01	0.29	< 0.01	0.18	ns	0.12	ns	0.42	< 0.01	0.32	< 0.01
Pubic hair appearance	0.08	ns	0.05	ns	0.50	< 0.01	0.46	< 0.01	0.24	0.04	0.20	ns	0.16	ns	0.26	0.02
Total energy intake	0.09	ns	0.05	ns	0.16	ns	0.15	ns	0.15	ns	0.22	ns	0.16	ns	0.07	ns
Salt intake	− 0.13	ns	− 0.17	ns	0.18	ns	0.19	ns	0.08	ns	0.09	ns	0.06	ns	0.00	ns
Exercise (e.g., jogging or sports)	0.06	ns	0.06	ns	0.08	ns	0.09	ns	0.08	ns	0.04	ns	− 0.01	ns	0.03	ns
Sedentary behavior (e.g., media use)	0.04	ns	0.10	ns	− 0.07	ns	− 0.06	ns	0.09	ns	− 0.03	ns	0.07	ns	0.15	ns
Whole body fat	0.42	< 0.01	0.47	< 0.01	− 0.12	ns	− 0.09	ns	0.10	ns	0.05	ns	0.25	0.02	0.01	ns
SBP	0.19	ns	0.13	ns	0.07	ns	0.05	ns	0.34	< 0.01	0.24	0.04	0.32	< 0.01	0.28	< 0.01
DBP	0.07	ns	0.05	ns	0.12	ns	0.10	ns	0.15	ns	0.18	ns	0.23	0.03	0.34	0.01

*N* number, *TAR* trunk-to-appendicular fat ratio, *TLR* trunk-to-leg fat ratio, *SBP* systolic blood pressure, *DBP* diastolic blood pressure, *ns* not significant

TAR was calculated as trunk fat divided by appendicular fat. TLR was calculated as trunk fat divided by leg fat. Pearson's correlation coefficient was used to evaluate relationships. *P* < 0.05 was considered statistically significant

Table 5 shows standardized partial regression coefficients between parameters of fat distribution at baseline and BP at follow-up. After adjusting for age, height, pubic hair appearance, whole body fat, and SBP at baseline, TAR was significantly related to SBP at follow-up in boys. Similarly, TLR in boys was significantly related to SBP at follow-up after adjusting for confounding factors including whole body fat and SBP at baseline.

**Table 5:** Relationships between fat distribution at baseline and blood pressure at follow-up

		TAR		TLR	
		*β*	*P*	*β*	*P*
Girls, *N* = 68	SBP	− 0.04	ns	− 0.04	ns
	DBP	0.02	ns	0.00	ns
Boys, *N* = 87	SBP	0.24	0.04	0.25	0.02
	DBP	0.04	ns	0.05	ns

*TAR* trunk-to-appendicular fat ratio, *TLR* trunk-to-leg fat ratio, *β* standardized partial regression coefficient, *N* number, *SBP* systolic blood pressure, *ns* not significant, *DBP* diastolic blood pressure

TAR was calculated as trunk fat divided by appendicular fat. TLR was calculated as trunk fat divided by leg fat. Multiple regression analysis was used after adjusting for age, height, pubic hair appearance, whole body fat, and SBP/DBP at baseline. *P* < 0.05 was considered statistically significant

**Fig. 1:**
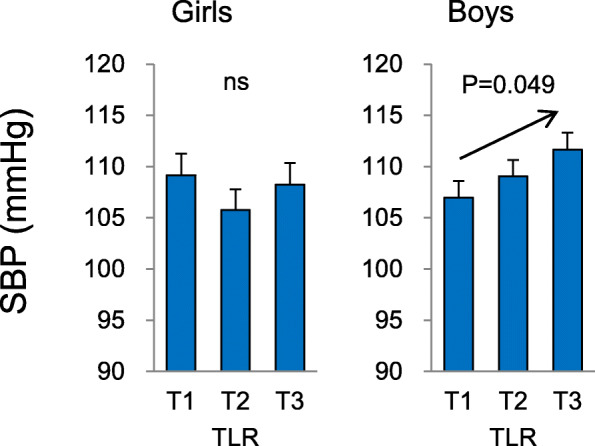
Adjusted means and standard errors of systolic blood pressure (SBP) at follow-up for tertiles (T1, T2, and T3) of trunk-to-leg fat ratio (TLR). Mean values were calculated from the general linear model after adjusting for confounding factors, including the age, height, pubic hair appearance, whole body fat, and SBP at baseline. Arrows show a significant trend of SBP from T1 to T3. For trend tests of SBP, multiple linear regression analysis was performed. The dependent variable was SBP at follow-up, and independent variables were tertiles of TLR, age, height, pubic hair appearance, whole body fat, and SBP at baseline

Figure 1 shows the adjusted mean of SBP at follow-up in each tertile of TLR at baseline. Adjusted means of SBP significantly increased from the lowest to highest tertiles of TLR in boys after adjusting for age, height, pubic hair appearance, whole body fat, and SBP at baseline.

## Discussion

Some individuals are not obese on the basis of height and weight, are insulin-resistant, predisposed to type 2 diabetes, and have premature coronary heart disease [2]. Such MONW individuals are very common in the general population [2]. The present cohort study targeting normal-weight pubertal children in the northeast region of Japan revealed that trunk-to-peripheral fat ratio in boys aged 9.6–12.6 years was positively associated with BP levels in boys aged 12.3–15.3 years. Thus, an excessive proportion of trunk fat rather than peripheral fat (i.e., fat with a more centralized distribution) was found to be associated with higher BP in boys of the present study, which also revealed that normal-weight boys with a more centralized fat distribution tended to have relatively high levels of BP later on. Interestingly, this relationship between fat distribution and BP was independent of whole body fat volume. Even among those who are not overweight or obese, more centralized fat distribution was associated with higher BP. It is well known that there is a certain degree of predictability in BP level when this is tracked from childhood to adulthood and that elevated BP levels persist over time and progress to adult hypertension [31]. Therefore, it is important to consider body fat distribution in addition to body fat mass to assess cardiometabolic risks [23]. The present results are consistent with previous cross-sectional and cohort studies from the central region of Japan (Hamamatsu City) [18, 22], and present and previous [18, 22] findings suggest that a large volume of body fat is not a prerequisite for developing high BP levels in childhood. Weight-independent parameters of body fat distribution measured by DXA provide additional information for evaluating cardiovascular risks and are particularly meaningful when assessing normal-weight boys.

Only a few studies have investigated whether relationships between fat distribution and BP are independent of those between fat volume and BP. In the present study, when parameters of fat distribution and amount of fat mass were included in the same multiple regression models, the relationship between fat distribution and BP was still significant. This suggests that the relationship between fat distribution and BP was independent of the relationship between whole body fat volume and BP. Similarly, one study found that the relationship between fat distribution and BP was independent of the relationship between whole body fat and BP in a cross-sectional study of Hamamatsu boys [18]. These findings suggest that not only the amount of fat but also the pattern of its distribution influence BP levels. This information may be particularly relevant for boys who have a normal amount of body fat that follows a more centralized distribution. The ratio of trunk-to-peripheral fat thus may be valuable for the evaluation of cardiovascular risks.

Values of TAR/TLR and BP stratified by age (Table 2) show significant increases with age. In addition, SBP levels in girls and boys show significant increases with age, suggesting that one parameter of growth (age) is a confounding variable that is associated with both the predictor (TAR/TLR) and the outcome (BP). The present study also revealed significant associations between TAR/TLR and growth parameters (age, height, pubic hair appearance), and between BP and parameters of growth (Table 4). This also suggests that growth is a confounding factor that modifies the association between TAR/TLR and BP. As such, we conducted multiple regression analyses after adjusting for confounding factors including age, height, and pubic hair appearance to assess the relationship between TAR/TLR and BP and identified several statistically significant associations that remained in boys after adjustments were made (Table 5, Fig. 1). Collectively, these findings indicate that there is an independent association between TAR/TLR and BP in addition to that between growth and blood pressure in boys.

Although we found that an increased ratio of trunk-to-peripheral fat was correlated with higher BP in boys, we observed no relationship between fat distribution and BP in girls. In other words, the relationship between fat distribution and BP differed by sex in the present study. A previous cross-sectional study of Hamamatsu children also reported a significant relationship between trunk-to-peripheral fat ratio and BP in boys but not in girls [18]. It is well known that sex affects fat deposit patterns and that women have more subcutaneous fat than men, while men have more visceral fat than women [32]. Visceral fat is thought to play a key role in the cluster of cardiometabolic abnormalities [33], and trunk fat includes both visceral and subcutaneous fat. Women, whose trunk fat tends to comprise less visceral fat than that in men, also exhibit a lower prevalence of obesity-related metabolic disorders than men [32]. Differences in the proportion of visceral fat in trunk fat between boys and girls might explain the effects of sex in the relationship between cardiovascular and body fat parameters. Another possible reason for the sex-dependent difference could be explained by the difference in growth, as the timing of secondary sexual characteristic development varies between girls and boys. Specifically, it is well known that pubertal changes in girls usually begin before those in boys of the same age. In Japanese children, the peak height velocity has been found to occur around the age of 11 years in girls and around 13 years in boys [34]. The difference in pubertal growth between girls and boys may modify the association between the trunk-to-peripheral fat ratio and blood pressure.

The strength of this study is that the analyzed population comprised 79.3% of the source population, which included almost all children residing in the Shiokawa area in Kitakata City; only 14.5% of baseline participants dropped out at follow-up. Moreover, no remarkable differences were observed between the follow-up population (analyzed population) and the drop-out population. Therefore, the effects of selection bias are smaller compared to our previous study in Hamamatsu City (i.e., the analyzed population was 48.8% of the source population and 61.5% of the baseline population) [22]. In addition, the present study obtained enough data on potential confounders, including dietary intake and exercise; from these potential confounding variables, we selected confounding factors for analysis. Thereafter, we assessed the relationships between exposure, outcomes, and confounding factors. Furthermore, the subject age in the present study spanned a wider range (9.6–12.6 years old at baseline and 12.3–15.3 years old at follow-up) than that of the previous study in Hamamatsu City.

However, the present study also has some limitations worth noting, including a potential selection bias. First, participants were enrolled from one area in one city in Japan, rather than from the entire country. However, according to standard growth charts of Japanese children based on national surveys, mean height/weight of boys at age 11.2 and girls at age 11.1 were 142.5 cm/36.6 kg and 143.5 cm/36.9 kg, respectively. Thus, there were no remarkable differences in anthropometric variables between the present followed-up population and the general population in Japan [35]. Second, childhood BMI rises with chronological age and biological age. To avoid misclassifying underweight or overweight children, age-specific cut-offs of BMI should be used to reflect the maturational tempo [36]. However, there are no age-specific cut-offs for BMI which reflect the impact of pubic hair appearance. Normal-weight children were identified using sex- and age-specific international cut-offs of BMI in the present study [26, 27].

## Conclusions

The present cohort study targeting community-dwelling children in the northeast region of Japan revealed that the trunk-to-peripheral fat ratio measured by DXA was associated with future BP in normal-weight boys. Normal-weight boys who had a more centralized distribution of fat tended to have relatively high levels of BP later on. This relationship between fat distribution and BP was independent of whole body fat volume. Our findings suggest that a large volume of body fat may not be a prerequisite for the development of high BP levels in childhood and that weight-independent indices such as trunk-to-peripheral fat ratio may be useful for predicting future BP. Thus, body fat distribution is a meaningful measure, particularly in the assessment of normal-weight boys.

## Data Availability

The datasets generated during the current study are not publicly available, but are available from the corresponding author on reasonable request.

## Abbreviations

BMI: Body mass index
BP: Blood pressure
DBP: Diastolic blood pressure
DXA: Dual-energy X-ray absorptiometry
FFQ: Food frequency questionnaire
HbA1c: Hemoglobin A1c
HDL-C: High-density lipoprotein cholesterol
LDL-C: Low-density lipoprotein cholesterol
MONW: Metabolically obese, normal-weight
SBP: Systolic blood pressure
TAR: Trunk-to-appendicular fat ratio
TLR: Trunk-to-leg fat ratio
T-C: Total cholesterol

## References

[1] Siervogel RM, Demerath EW, Schubert C, Remsberg KE, Chumlea WC, Sun S (2003). Puberty and body composition. Horm Res..

[2] Ruderman N, Chisholm D, Pi-Sunyer X, Schneider S (1998). The metabolically obese, normal-weight individual revisited. Diabetes..

[3] Ruderman NB, Schneider SH, Berchtold P (1981). The “metabolically-obese”, normal-weight individual. Am J Clin Nutr..

[4] Tchernof A, Despres JP (2013). Pathophysiology of human visceral obesity: an update. Physiol Rev..

[5] Koh H, Hayashi T, Sato KK, Harita N, Maeda I, Nishizawa Y (2011). Visceral adiposity, not abdominal subcutaneous fat area, is associated with high blood pressure in Japanese men: the Ohtori study. Hypertens Res..

[6] Tsushima H, Yamamoto H, Kitagawa T, Urabe Y, Tatsugami F, Awai K (2015). Association of epicardial and abdominal visceral adipose tissue with coronary atherosclerosis in patients with a coronary artery calcium score of zero. Circ J..

[7] Staiano AE, Gupta AK, Katzmarzyk PT (2014). Cardiometabolic risk factors and fat distribution in children and adolescents. J Pediatr..

[8] Krotkiewski M, Bjorntorp P, Sjostrom L, Smith U (1983). Impact of obesity on metabolism in men and women. Importance of regional adipose tissue distribution. J Clin Invest..

[9] Larsson B, Svardsudd K, Welin L, Wilhelmsen L, Bjorntorp P, Tibblin G (1984). Abdominal adipose tissue distribution, obesity, and risk of cardiovascular disease and death: 13 year follow up of participants in the study of men born in 1913. Br Med J (Clin Res Ed).

[10] Czernichow S, Kengne AP, Stamatakis E, Hamer M, Batty GD (2011). Body mass index, waist circumference and waist-hip ratio: which is the better discriminator of cardiovascular disease mortality risk?: evidence from an individual-participant meta-analysis of 82 864 participants from nine cohort studies. Obes Rev..

[11] Coutinho T, Goel K, Correa de Sa D, Kragelund C, Kanaya AM, Zeller M (2011). Central obesity and survival in subjects with coronary artery disease: a systematic review of the literature and collaborative analysis with individual subject data. J Am Coll Cardiol..

[12] Albanese CV, Diessel E, Genant HK (2003). Clinical applications of body composition measurements using DXA. J Clin Densitom..

[13] Novotny R, Daida YG, Grove JS, Le Marchand L, Vijayadeva V (2006). Asian adolescents have a higher trunk: peripheral fat ratio than Whites. J Nutr..

[14] Zillikens MC, Uitterlinden AG, van Leeuwen JP, Berends AL, Henneman P, van Dijk KW (2010). The role of body mass index, insulin, and adiponectin in the relation between fat distribution and bone mineral density. Calcif Tissue Int..

[15] Savgan-Gurol E, Bredella M, Russell M, Mendes N, Klibanski A, Misra M (2010). Waist to hip ratio and trunk to extremity fat (DXA) are better surrogates for IMCL and for visceral fat respectively than for subcutaneous fat in adolescent girls. Nutr Metab (Lond).

[16] Ackerman KE, Davis B, Jacoby L, Misra M (2011). DXA surrogates for visceral fat are inversely associated with bone density measures in adolescent athletes with menstrual dysfunction. J Pediatr Endocrinol Metab..

[17] Morimoto Y, Maskarinec G, Conroy SM, Lim U, Shepherd J, Novotny R (2012). Asian ethnicity is associated with a higher trunk/periphery fat ratio in women and adolescent girls. J Epidemiol..

[18] Kouda K, Nakamura H, Fujita Y, Ohara K, Iki M (2012). Increased ratio of trunk to appendicular fat and increased blood pressure: study of a general population of Hamamatsu children. Circ J..

[19] Peppa M, Koliaki C, Hadjidakis DI, Garoflos E, Papaefstathiou A, Katsilambros N (2013). Regional fat distribution and cardiometabolic risk in healthy postmenopausal women. Eur J Intern Med..

[20] Kouda K, Nakamura H, Ohara K, Fujita Y, Iki M (2015). Increased ratio of trunk-to-appendicularfat and decreased adiponectin: a population-based study of school children in Hamamatsu, Japan. J Clin Densitom..

[21] Kouda K, Dongmei N, Tamaki J, Iki M, Tachiki T, Kajita E (2017). Relative importance of central and peripheral adiposities on cardiometabolic variables in females: a Japanese Population-Based Study. J Clin Densitom..

[22] Kouda K, Ohara K, Fujita Y, Nakamura H, Iki M (2016). Trunk-to-peripheral fat ratio predicts subsequent blood pressure levels in pubertal children with relatively low body fat: three-year follow-up study. Circ J..

[23] Kishimoto I (2016). Trunk-to-leg fat ratio: an emerging early marker of childhood adiposity, and future cardiometabolic risks. Circ J..

[24] Tamaki J, Ikeda Y, Morita A, Sato Y, Naka H, Iki M (2008). Which element of physical activity is more important for determining bone growth in Japanese children and adolescents: the degree of impact, the period, the frequency, or the daily duration of physical activity?. J Bone Miner Metab..

[25] Kouda K, Fujita Y, Sato Y, Ohara K, Nakamura H, Uenishi K (2014). Fat mass is positively associated with bone mass in relatively thin adolescents: data from the Kitakata Kids Health Study. Bone..

[26] Cole TJ, Bellizzi MC, Flegal KM, Dietz WH (2000). Establishing a standard definition for child overweight and obesity worldwide: international survey. BMJ..

[27] Cole TJ, Flegal KM, Nicholls D, Jackson AA (2007). Body mass index cut offs to define thinness in children and adolescents: international survey. BMJ.

[28] Laskey MA (1996). Dual-energy X-ray absorptiometry and body composition. Nutrition..

[29] National High Blood Pressure Education Program Working Group on High Blood Pressure in Children and Adolescents (2004). The fourth report on the diagnosis, evaluation, and treatment of high blood pressure in children and adolescents. Pediatrics.

[30] Uenishi K, Ishida H, Nakamura K (2008). Development of a simple food frequency questionnaire to estimate intakes of calcium and other nutrients for the prevention and management of osteoporosis. J Nutr Sci Vitaminol (Tokyo)..

[31] Bao W, Threefoot SA, Srinivasan SR, Berenson GS (1995). Essential hypertension predicted by tracking of elevated blood pressure from childhood to adulthood: the Bogalusa Heart Study. Am J Hypertens..

[32] Nedungadi TP, Clegg DJ (2009). Sexual dimorphism in body fat distribution and risk for cardiovascular diseases. J Cardiovasc Transl Res..

[33] Kishida K, Funahashi T, Matsuzawa Y, Shimomura I (2012). Visceral adiposity as a target for the management of the metabolic syndrome. Ann Med..

[34] Tanaka T, Suwa S, Yokoya S, Hibi I (1988). Analysis of linear growth during puberty. Acta Paediatr Scand Suppl..

[35] Suwa S, Tachibana K (1993). Standard growth charts for height and weight of Japanese children from birth to 17 years based on cross-sectional survey of national data. Clin Pediatr Endocrinol..

[36] Mumm R, Scheffler C, Hermanussen M (2014). Developing differential height, weight and body mass index references for girls that reflect the impact of the menarche. Acta Paediatr..

